# Invertebrate Communities and Driving Factors Across Woody Debris Types in Temperate Forests, Northern China

**DOI:** 10.3390/biology15010043

**Published:** 2025-12-26

**Authors:** Jinkai Dong, Zhiwei Qi, Mingliang Cao, Zijin Wang, Xueqian Ji, Jinyu Yang

**Affiliations:** College of Forestry, Hebei Agriculture University, Baoding 071001, China; 15532781108@163.com (J.D.); qizhiwei0722@163.com (Z.Q.); c2872523725@163.com (M.C.); 18632211679@163.com (Z.W.); 15066036631@163.com (X.J.)

**Keywords:** woody debris, invertebrates, decay stage, tree species, forest types

## Abstract

Woody debris decomposition is a key process in forest ecosystem material cycles, with invertebrate communities playing a vital role. Distinct physicochemical properties of woody debris types lead to varying effects on these communities. Taking woody debris in Saihanba’s *Larix principis-rupprechtii* plantations, *Betula platyphylla* natural secondary forests, and larch–birch mixed forests (northern China) as objects, we collected woody debris-inhabiting invertebrates via hand-sorting. We studied how tree species (larch/birch), forest types (pure/mixed), and decay stages (I–V) collectively regulate invertebrate community assembly. Results showed significant differences in woody debris physicochemical properties across these factors. Phytophagous groups dominated early decay stages (I–III) and decreased significantly (*p* < 0.05) with reduced wood density. In contrast, saprophagous and predatory groups increased with decay, correlated with higher TN and were more abundant in mixed than pure forests. NMDS indicated significant community differences among tree species/forest types in early decay, converging later. PLS-PM further confirmed functional groups’ response pathways to woody debris characteristics. Thus, preserving woody debris integrity and diversity in plantations is crucial for maintaining invertebrate diversity, promoting nutrient cycling, and enhancing forest ecosystem functions.

## Simple Summary

In forest ecosystems, fallen trees (woody debris) function as important shelters rather than waste, providing food and shelter for diverse invertebrates. Our study demonstrates that invertebrate community composition undergoes systematic succession as wood decay progresses: phytophagous groups (e.g., cerambycid beetles) dominate initial decay stages while saprophagous (e.g., Diptera larvae) and predatory groups (e.g., centipedes) prevail in middle-to-late stages. Tree-species identity and forest type significantly influence invertebrate distribution. For instance, *Betula platyphylla* (birch) supports higher invertebrate abundance than *Larix principis-rupprechtii* (larch) due to its lower wood density and richer nutrient content. Mixed forests enhance this diversity by supplying heterogeneous woody debris substrates and creating complex microhabitats, thereby sustaining more intricate food webs compared to pure types. This study indicates that the conservation of fallen dead trees of various tree species and at different decay stages in forests are of great significance for maintaining soil biodiversity, promoting nutrient cycling, and enhancing the health and sustainability of forest ecosystems.

## 1. Introduction

Woody debris plays a critically important role in complex forest ecosystems. As organic matter remaining after the natural death of trees, woody debris serves not only as an ecological cornerstone of the forest floor but also as a habitat and food source for numerous organisms [1,2]. Woody debris fulfills a central function in material cycling and energy flow as it releases nutrients usable by microorganisms and other organisms through the decomposition process, thereby promoting the health and stability of the forest ecosystem. In this process, invertebrates are key participants, playing a significant role in the decomposition of woody debris and nutrient cycling [3].

Although forest detrital systems are species-rich, studies on the dynamics of invertebrate communities within woody debris remain relatively scarce [4]. However, understanding of colonization by invertebrates and their community composition associated with woody debris decomposition is even more limited. These organisms accelerate the decomposition of woody debris through various activities such as feeding, burrowing, and reproducing within it, thereby effectively releasing nutrients [5,6,7]. In the study by Zuo et al. [8], which focused on two saprophagous invertebrate groups—Diplopoda (millipedes) and Isopoda (woodlice)—it was found that as woody debris decomposed and wood density gradually decreased, the species abundance significantly increased, thereby effectively accelerating decomposition. Similarly, a study by Seibold et al. [9] demonstrated that the beta diversity of saproxylic beetle communities remained consistently high during the initial eight years of succession, increasing with greater phylogenetic distance, spatial separation, and forest structural differences, while the influence of phylogenetic distance weakened temporally. These studies indicate that invertebrates play important yet dynamic roles across various stages of woody debris decomposition, influencing material cycling and nutrient release within ecosystems. When living trees fall and form woody debris, it gradually provides a dynamic habitat that attracts colonization and activity of invertebrates. As woody debris decomposes, invertebrate diversity tends to increase. It is found that saproxylic beetles have higher diversity in the early decay stages, while oribatid mites and springtails reach diversity peaks in the mid-decay stages [10,11]. The traits of woody debris undergo gradual change over time (i.e., at different decay stages), leading to continuous alterations in the habitats of invertebrates [12,13]. Changes in habitat conditions, in turn, trigger succession within the associated biological communities, prompting invertebrates to exhibit distinct adaptive resource utilization strategies and ecological functions [14,15].

The composition of invertebrate communities is shaped not only by the decay stage of woody debris but also significantly by tree species specificity. Different tree species possess unique physicochemical properties, such as bark morphology, wood density, nutritional composition, and secondary metabolites [16]. For instance, one study found that the smoother bark of *Betula platyphylla*, compared to *Picea asperata*, directly affected the habitat selection by saprophagous invertebrates. Furthermore, differences in wood density, moisture content, pH, nutrient elements (e.g., nitrogen, phosphorus, potassium), and secondary metabolites between these two tree species resulted in a higher abundance of saprophagous invertebrates in *P. asperata* [8]. Another study observed that as woody debris transitions from the early to advanced decay stages, the structure and chemical composition of the wood converge, leading to a corresponding convergence in the composition of invertebrate communities among different tree species during the late decomposition stages [17]. This phenomenon suggests an interactive effect between decay stage and tree species, collectively shaping the composition and function of invertebrate communities. Therefore, the diversity of tree species and the progression of decay stages jointly determine the dynamic characteristics of invertebrate communities and their functional roles within forest ecosystems [18].

Forest type (e.g., pure or mixed forests) significantly influences the physicochemical properties, spatial distribution, and subsequent decomposition process of woody debris through differences in tree species composition, forest structure, and microhabitat characteristics [19]. In contrast to pure forests, mixed forests typically consist of multi-species assemblages (e.g., coniferous and broad-leaved trees), forming more complex bark structures, litter layers, and micro-topographies, thereby providing a richer diversity of substrate types and refugia, which offer heterogeneous habitat conditions for invertebrates. Such differences in habitat structure not only enhance the species richness of invertebrates but also increase the compositional diversity and trophic complexity of the communities [20]. Owing to higher tree species diversity, mixed forests can continuously supply woody debris at different decay stages, thus attracting various functional feeding groups of invertebrate, including saprophagous and predatory taxa. Research indicates that such habitats help maintain higher beta diversity, meaning the differences in invertebrate communities among microhabitats become more pronounced with varying forest types [21]. For instance, a study in German forest systems showed that the presence of standing deadwood in mixed forests significantly increased beetle species richness and abundance, and their community composition also differed markedly depending on forest management practices [22]. These findings demonstrate that forest type, by regulating the type, quantity, and distribution of woody debris, directly affects the resource utilization patterns and community assembly processes of invertebrates [23].

In traditional forest management, woody debris has often been perceived as a potential trigger for forest fires and pest outbreaks, particularly within management paradigms oriented towards timber production in plantation forests, where complete removal is a frequently adopted measure [24,25]. However, this practice overlooks the irreplaceable ecological functions of woody debris in maintaining soil fertility, promoting biodiversity, and supporting ecosystem food webs [26]. Given that China possesses the world’s largest area of plantation forests with extensive coverage, enhancing ecosystem stability and service functions through scientific management has emerged as a critical and urgent research priority.

Against this backdrop, the present study focuses on three temperate forest types—*Larix principis-rupprechtii* forests, *Betula platyphylla* forests, and mixed larch–birch forests—to investigate the colonization characteristics of invertebrates in woody debris across different forest types and decay stages. To address this scientific question, we propose the following two hypotheses: First, we hypothesize that tree species identity and the decay stage of woody debris significantly influence the colonization characteristics of invertebrates under different forest type contexts. Specifically, the abundance and taxonomic composition of invertebrate communities will exhibit significant variations attributable to the differences in decay stage and tree species of the woody debris. Second, we further hypothesize that the physicochemical properties of woody debris serve as the key mediating variables linking tree species, forest types, and decay stages to the invertebrate community. Changes in these properties can drive the succession of invertebrate functional groups. Therefore, we aim to elucidate the interrelationships between the physicochemical properties of woody debris across different decay classes and the colonization characteristics of invertebrates among various forest types, thereby providing a deeper understanding of the crucial role that invertebrates play in forest ecosystem functioning.

## 2. Materials and Methods

### 2.1. Study Area

This study was conducted in the Saihanba Mechanical Forest Farm (42°02′–42°36′ N, 116°51′–117°39′ E; elevation 1010–1939.9 m a.s.l.) located in Chengde City, Hebei Province, China. The forested area of the region spans approximately 76,700 hectares, with a total timber volume of about 10.368 million cubic meters. The climate is characterized as a cold-temperate continental monsoon climate zone, featuring semi-arid to semi-humid conditions. The mean annual temperature is −1.5 °C, the annual average precipitation is 467 mm, predominantly concentrated between June and August, the annual evaporation is 230 mm, the annual sunshine duration totals 2368 h, and the average frost-free period is 64 days. The forest coverage rate reaches approximately 82.6%. Dominant tree species include *Larix principis-rupprechtii*, *Betula platyphylla*, *Picea asperata*, and *Pinus sylvestris var. mongolica*, among others.

### 2.2. Tree Species and Forest Type Selection

The study was conducted in the Saihanba Mechanical Forest Farm from July to August 2023. The selected tree species, *L. principis-rupprechtii* (larch) and *B. platyphylla* (birch), hold a dominant ecological status and considerable management value in this region. Meanwhile, these two species exhibit marked differences in key physical traits, such as bark thickness, surface roughness, and wood hardness.

**Figure 1:**
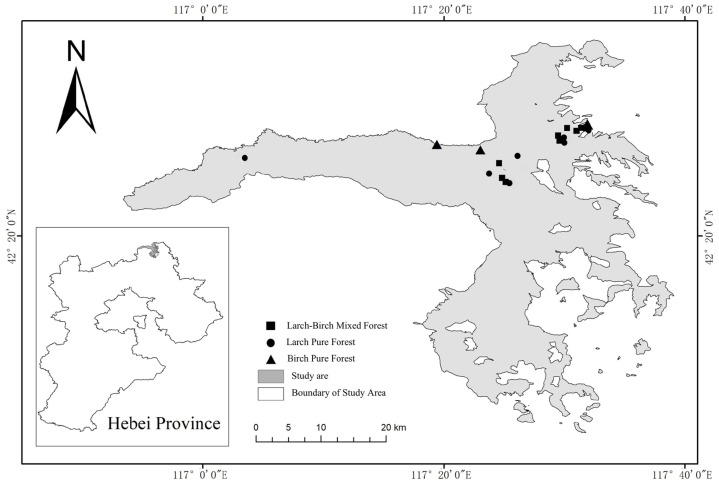
Nineteen sampling sites with woody debris across three forest types (six larch pure forests, three birch pure forests, and ten larch–birch mixed forests) and various decay stages considered in the present study in Saihanba.

The investigation focused on three forest types: pure larch forests, pure birch forests, and mixed larch–birch forests. All sample plots were established in areas with similar site conditions and uniformly consistent growth environments. Each plot covered an area of at least 10 hectares, with adjacent plots spaced a minimum of 2 km apart. All stands were over 40 years old and aside from intermediate thinning conducted during the sapling-to-pole stage, no further management interventions had been implemented. Consequently, the plots contained a substantial amount of woody debris. A total of 19 sample plots were established for the study (Figure 1), the basic information about the sample plots and sample trees is displayed in Table 1.

### 2.3. Collection of Woody Debris and Soil Samples

All sampled woody debris was ground-contact logs. The decay degree of woody debris was assigned based on the classification criteria established by Spies et al. (1988) and Rouvinen et al. (2002), with five decay classes from fresh to highly decomposed: I, II, III, IV, and V [27,28]. Woody debris was categorized by forest type as follows: woody debris from larch pure forest (LP), birch pure forest (BP), larch mixed forest (LM), and birch mixed forest (BM), where in both LM and BM are two distinct types of woody debris collected from mixed larch–birch forests. To minimize errors, woody debris was collected away from ditches and ant nests, and additional efforts were made to sample multiple logs across different tree species, forest types, decay classes, and diameter classes (large diameter ≥ 10 cm; fine woody debris: 2.5–10 cm) [29] to ensure uniform sample size distribution. A 50 cm middle segment was excised from each collected log for subsequent experiments. A total of 168 woody debris segments were initially collected. After excluding samples contaminated by ant nests or affected by other disturbances, 147 segments were retained for the final analysis. From each segment, a 5 cm thick disk was sawn for measuring physicochemical properties. For highly decomposed debris (decay classes IV and V), a core sampler of known volume was used for sampling, and the remaining portions were reserved for invertebrate collection.

Under the in situ location of each collected woody debris, the litter layer was removed, and two sampling points were randomly selected. A soil auger with a diameter of 5 cm was used to drill and collect surface soil at the 0–10 cm depth, which were designated as soil samples under woody debris. In each plot, 10 sampling points far away from woody debris (>2 m) were randomly selected for soil sampling, which were designated as plot soil samples. All the above soil samples were placed into sealed plastic bags, thoroughly mixed, and properly labeled.

### 2.4. Invertebrate Collection

The woody debris was placed in a tray to prevent invertebrates escaping. Bark attached to the debris was carefully removed with forceps, and the wood was fragmented using knives or hatchets to ensure complete collection of all invertebrates (both adult and larval) associated with the woody debris. All collected specimens were preserved in 95% ethanol for identification and counting. Specimens were examined under a Nikon SMZ-745 stereomicroscope, and identification was performed using specialized taxonomic keys [30]. Invertebrates were identified to the species level whenever possible; those that could not be determined at this level were classified into morphospecies (e.g., sp1, sp2, sp3…) at the family level.

### 2.5. Determination of Physicochemical Properties of Woody Debris and Soils

The 5 cm thick disks sawn from the woody debris were transported to the laboratory for determination of their physicochemical properties. The density of the woody debris was measured via the water displacement method: Each disk was first weighed to record its wet weight and then completely immersed in a graduated cylinder containing distilled water. The volume of displaced water was recorded as the volume of the disk. The disk was then oven-dried at 65 °C until a constant weight was achieved, and its dry weight was measured. The density of the woody debris was calculated as the dry weight of the disk divided by its volume. The wood moisture content was calculated as (wet weight − dry weight)/wet weight.

The dried disks were ground to a homogeneous powder using a mechanical grinder. The total nitrogen (TN) and total carbon (TC) contents of the woody debris were determined using an elemental analyzer. The lignin content was measured using a Lignin Content Assay Kit (Kit No.: BC4205), and the cellulose content was determined using a Cellulose (CLL) Content Assay Kit (Kit No.: BC4285), both obtained from Beijing Solarbio Science & Technology Co., Ltd, Beijing, China.

Soil samples were placed in a 65 °C oven and dried to constant weight. After passing through a 100-mesh sieve, the total nitrogen (TN) and total carbon (TC) contents were determined using an elemental analyzer.

### 2.6. Data Analysis

The number of invertebrates collected from woody debris across various tree species, forest types, and decay classes was utilized to calculate the invertebrate individual density (ind/kg), the Shannon–Wiener diversity index, and the Pielou evenness index. Taxonomic groups were classified based on their relative abundance within the total community collection according to a tiered system: groups constituting more than 10.0% of the total abundance were classified as dominant groups; those representing 1.0% to 10.0% were classified as common; and those accounting for less than 1.0% were classified as rare [31,32]. The calculation of the Shannon–Wiener diversity index was not feasible for some woody debris samples from which no invertebrates were collected, leading to a lack of necessary data support. Furthermore, the mathematical assessment of the Pielou evenness index is undefined for samples containing only a single species or entirely lacking invertebrates. Due to these specific data integrity requirements for ecological metrics, samples that did not meet the criteria for the respective indices were excluded from the corresponding statistical modeling processes in this study.

One-way analysis of variance (ANOVA) was employed to test for differences in the physicochemical properties of the woody debris. The non-parametric Kruskal–Wallis test was used to examine differences in dominant invertebrate groups among different woody debris types. A multi-way ANOVA was applied to assess the effects of tree species, forest type, and decay class on the diversity characteristics of invertebrates within the woody debris. Non-metric Multidimensional Scaling (NMDS) based on Bray–Curtis distance was used to visualize invertebrate community composition dissimilarities; NMDS, PERMANOVA, ANOSIM, envfit, and pairwise comparisons of decay classes were implemented via the vegan package in R 4.4.1. Data processing was conducted with the dplyr package in R 4.4.1, and NMDS ordination plots were generated using the ggplot2 package in R 4.4.1. Bubble plots were utilized to illustrate the variations in individual density of dominant invertebrate groups across different tree species, forest types, and decay classes of woody debris. Path analysis was conducted using a partial least squares path model (implemented in Smart PLS software, version 4.0). Data organization was performed using Microsoft Excel 2016. Descriptive statistical analyses were carried out with SPSS 24. All graphs were plotted using the ggplot2 package in R software (version 4.4.1).

## 3. Results

### 3.1. Physicochemical Properties of Woody Debris

Among different forest types within the same decay class (Table 2), in the early decay stage (class I) the wood moisture content of *B. platyphylla* was significantly higher than that of *L. principis-rupprechtii* in both pure and mixed forests (*p* < 0.05). Regarding wood density, from decay class I to IV the density of larch woody debris in pure forests was significantly higher than that of birch (*p* < 0.05). The total nitrogen (TN) content of woody debris in mixed forests was higher than in pure stands, while the carbon-to-nitrogen (C:N) ratio was lower. These differences were statistically non-significant.

Among different decay classes within the same forest stand: The moisture content of woody debris increased with the advancement of decay classes in all forest types, though the increase was not significant, and peaked at decay class V. In terms of woody debris density, it was relatively high in the early stages of decomposition and gradually decreased in the later stages. This difference was most significant in larch (*p* < 0.05). TN content increased with the progression of decay classes, with the most marked increase observed in the late decomposition stages of mixed forests. In contrast, the carbon-to-nitrogen ratio decreased as decay classes advanced (*p* < 0.05). Lignin content, cellulose content and total carbon (TC) content remained relatively stable across all decomposition stages.

### 3.2. Overview of Invertebrate Survey

A total of 4312 invertebrate individuals were collected from the woody debris, belonging to 5 classes, 14 orders, 59 families, and 88 taxonomic groups (Table 3). The dominant groups were Lithobiidae, Scolytidae, and Chironomidae, comprising 1551 individuals and accounting for 35.97% of the total abundance. Common groups included Staphylinidae, Tipulidae, and Elateridae, among others, with 2155 individuals, representing 49.98% of the total. Rare groups, such as Tubificidae, Pseudoscorpionidae, Siricidae, and Carabidae, constituted 606 individuals, accounting for 14.05% of the total collection.

### 3.3. The Effects of Tree Species, Forest Type, and Decay Class of Woody Debris on Invertebrate Diversity

A multi-way analysis of variance indicated that tree species, forest types, and decay classes of woody debris had significant effects on the characteristics of invertebrate communities. Specific results are as follows: The individual density of invertebrates in birch woody debris was significantly higher than that in larch woody debris (F = 8.792, *p* < 0.01). In decay class II, the Shannon–Wiener diversity index of invertebrates in pure birch forests was significantly lower than that in other forest types (F = 7.407, *p* < 0.01). Both the individual density and taxon richness of invertebrates in all woody debris samples increased with the advancement of decay classes (F = 4.689, *p* < 0.01). In addition, the interaction between tree species and decay classes exerted significant effects on both the Shannon–Wiener diversity index (F = 2.883) and the taxon richness (F = 2.868) of invertebrates in woody debris, but the significance only reached the 0.05 level (Table 4, Figure S1).

### 3.4. Distribution Characteristics of Dominant Invertebrate Groups in Woody Debris

**Figure 2:**
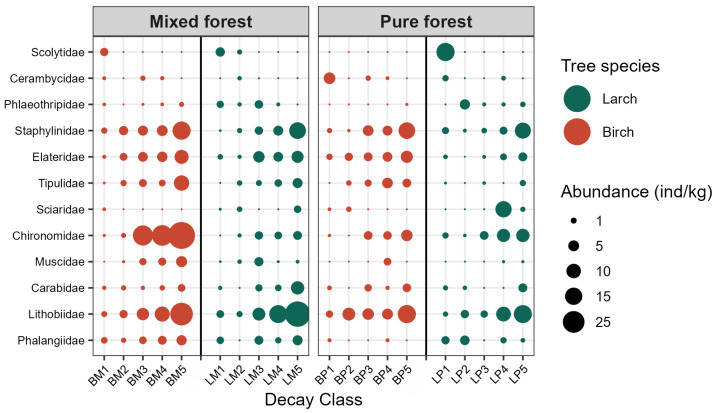
The abundance of dominant invertebrate taxa in woody debris. Species with higher abundance in each woody debris types were categorized into three trophic groups (top to bottom): Phytophagous (Scolytidae, Cerambycidae, Phlaeothripidae), Saprophagous (Staphylinidae, Elateridae, Tipulidae, Sciaridae, Chironomidae, Muscidae), and Predatory (Carabidae, Lithobiidae, Phalangiidae).

Comparison of the abundance and distribution of dominant invertebrate groups (at the family level) in woody debris between pure and mixed forests across different decay classes (Figure 2) revealed that the phytophagous functional group exhibited the highest abundance in decay classes I, II, and III, with a significant decline in abundance as decay class advanced, particularly in Scolytidae and Cerambycidae (*p* < 0.05) (Table S1). In contrast, saprophagous invertebrates showed an opposite trend to phytophagous groups, with abundance increasing significantly with progressive decay stages, most notably in Tipulidae, Chironomidae, and Staphylinidae (*p* < 0.05). The abundance of predatory invertebrates followed a pattern similar to saprophagous groups, as observed in Lithobiidae and Phalangiidae, which increased with decay class and peaked at class V. Furthermore, for the same tree species, the total abundance of phytophagous invertebrates in woody debris was higher in pure forests than in mixed forests, whereas the abundance of saprophagous and predatory invertebrates was significantly greater in mixed forests.

### 3.5. Distribution of Invertebrates in Woody Debris Based on NMDS Analysis

**Figure 3:**
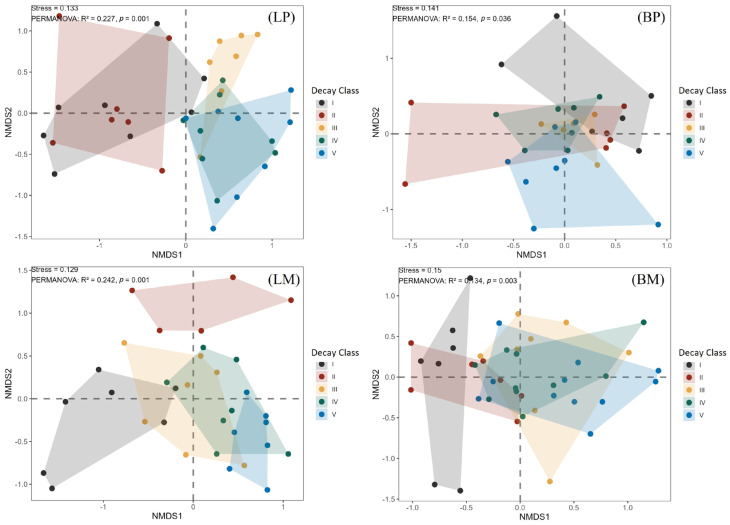
NMDS ordination by decay stage. Panels show different woody debris types: LP, BP, LM, and BM. Points are colored by decay stage: I (black), II (red), III (yellow), IV (green), and V (blue), with corresponding translucent polygons indicating group dispersion. Stress values (ranging from 0.129 to 0.150) reflect a good ordination fit.

Non-metric Multidimensional Scaling (NMDS) results revealed that invertebrate communities in the four woody debris types (BM, BP, LM, LP) exhibited significant differences among different forest types during the early decay stages (*p* < 0.05) (class I and II). As the decay class advanced to the late stages (class III–V), the structures of these communities gradually converged (*p* > 0.05) (Figure 3).

**Figure 4:**
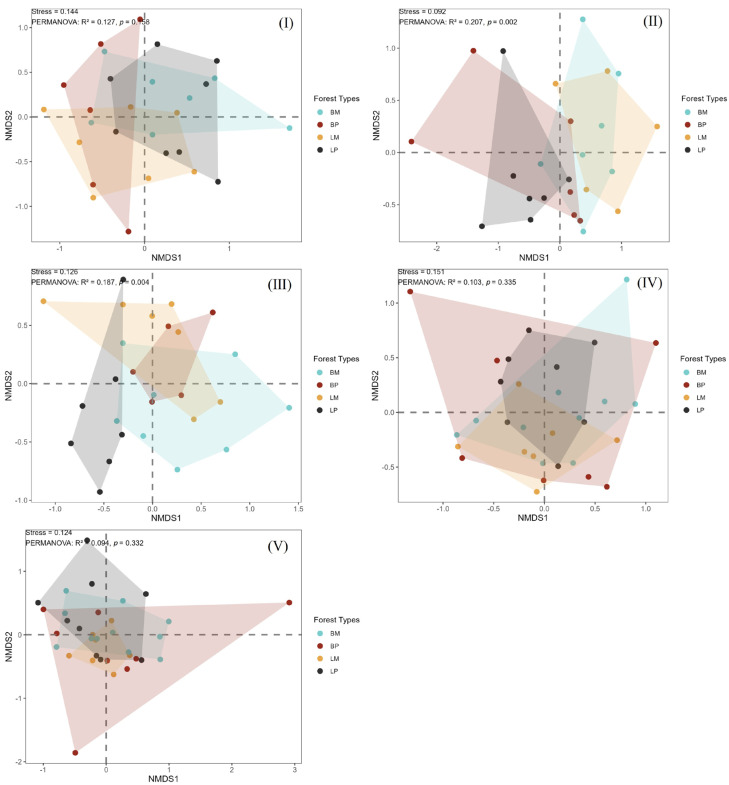
NMDS ordination by forest type. Points correspond to woody debris: BM (blue), BP (red), LM (yellow), and LP (black). In the subfigure, I−V represent decay classes I−V. Polygons enclose invertebrate communities from their respective forest types. Stress values range from 0.092 to 0.151, indicating good ordination reliability.

This result was consistent with the NMDS analysis grouped by decay classes (Figure 4). Specifically, the community structures in the early decay stages (class I and III) were significantly separated from those in the late stages (class IV−V) across different forest types (*p* < 0.05), while the sample points within classes IV−V showed obvious clustering (*p* > 0.05). This phenomenon reflected that with the progression of the decomposition process, microhabitats and resource conditions converged, leading to a convergent trend in the composition of invertebrate communities.

In conclusion, the invertebrate communities in woody debris were significantly affected by tree species and forest types during the early decay stages, whereas they gradually converged with the intensification of decomposition in the middle and late stages.

### 3.6. Interrelationships Between Woody Debris and Invertebrates

**Figure 5:**
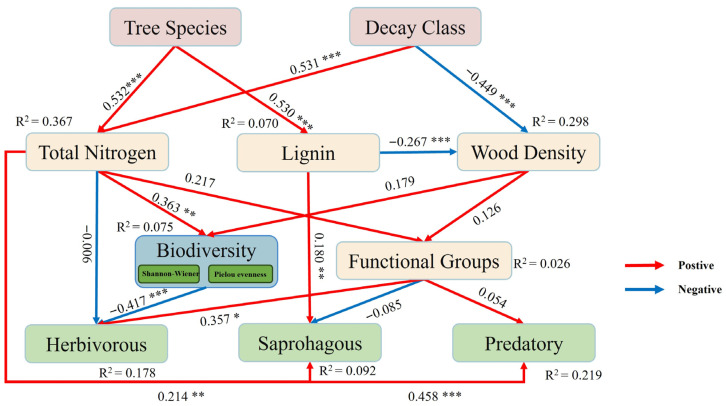
PLS−PM path diagram of effects of woody debris on invertebrate communities and functional groups. Standardized path coefficients are indicated on the arrows (red = positive effect; blue = negative effect), with R^2^ values shown for all latent variables. *p* < 0.05: *; *p* < 0.01: **; *p* < 0.001: ***.

The Partial Least Squares Path Modeling (PLS−PM) was used to elucidate the causal relationships between tree species, decay class, physicochemical properties of woody debris, and invertebrate communities (Figure 5). The model results showed that decay class and tree species were the key factors driving changes in the physicochemical properties of woody debris. Both factors exerted a highly significant negative direct effect on the TN content of wood, compared with lignin content (R^2^ = 0.070), total nitrogen (TN) in wood (R^2^ = 0.367) had stronger predictive power for invertebrate community variables. Lignin content only exerted a significant positive effect on saprophagous invertebrates (path coefficient = 0.18, *p* < 0.01). The paths from TN to the three trophic groups of invertebrates further highlight its dominant role in shaping invertebrate community structure during the wood decomposition process. Additionally, decay class also exhibited a highly significant positive direct effect on wood density, thereby indirectly influencing invertebrate communities (path coefficient = −0.449, *p* < 0.001). Tree species had a highly significant effect on lignin content, which further indirectly impacted wood density and the density of saprophagous species (path coefficient = 0.53, *p* < 0.001).

## 4. Discussion

Although plantation forests make remarkable contributions to the expansion of global forest resources, they generally suffer from issues such as low tree species composition and simplified stand structure. These problems result in weak ecosystem stability, severe soil fertility degradation, and reduced biodiversity, thereby impairing the ecosystem functions of material cycling and energy flow [33,34]. Woody debris, as a vital component of forest ecosystems, plays an especially crucial role. Our research found that soil total nitrogen (TN) content under woody debris was higher than that in soil without woody debris coverage, and it gradually increased as woody debris decomposed. Furthermore, regardless of the presence of woody debris, soil TN and total carbon (TC) were higher in mixed forests than in pure forests (Table A1). During the decomposition of woody debris, detritus generated by fragmentation, solubilization, and respiration processes enters the soil, thereby enhancing soil nutrient levels [15]. Additionally, under precipitation, woody debris releases part of the dissolved organic matter through leaching, which stimulates microbial activity [35,36]. Therefore, as a vital component of forest ecosystems, woody debris can effectively ameliorate the soil environment, provide heterogeneous habitats, and promote nutrient cycling as well as the colonization and succession of invertebrate communities. Tree species, forest types, and decay stages collectively regulated the dynamics of the physicochemical properties of woody debris [37,38,39]. This study found that at decay classes I and II, the moisture content and TN of birch woody debris were higher than those of larch woody debris, while its C:N and wood density were lower (Table 2). This discrepancy arises because, during the initial decomposition stage, birch features a dense, non-shedding bark structure that enables efficient water retention, resulting in a significantly higher moisture content and a substantially lower wood density relative to larch [40]. As decay class advanced, the moisture content of woody debris from both tree species exhibited an increasing trend, while the wood density continued to decline [41,42]. This change led to a gradual reduction in differences in moisture content and wood density between birch and larch debris, indicating that the homogenization process during decomposition progressively overrode the initial characteristics imposed by tree species and forest types, thereby driving the convergence of microenvironmental conditions and resource availability across different woody debris types.

The composition and functional groups of invertebrate communities in woody debris affirming previous studies exhibited significant variations across forest types and tree species (Figure 2). These distinctions primarily originate from the persistence of physicochemical properties in woody debris during early to mid-decay stages, which remain comparable to those of recently fallen logs. Freshly deposited woody debris, analogous to living trees, emits volatile organic compounds that attract specialized phytophagous invertebrates, leading to their preferential colonization of less decomposed material [43,44]. For instance, during the initial decay stages, pronounced differences in bark characteristics, wood density, and TN content between birch and larch give rise to distinct invertebrate assemblages demonstrating clear preferences for specific tree species and decay stages, as evidenced by cerambycid and scolytid beetle distributions. As decomposition advances beyond stage III, progressive bark detachment, substantial reductions in wood density, and microenvironmental homogenization occur. Consequently, phytophagous invertebrate abundance declines markedly, while saprophagous invertebrates (e.g., Diptera) become increasingly involved in decomposition processes, accompanied by rising predatory invertebrate abundance [45,46] until complete wood breakdown. This study further revealed consistently higher abundances of saprophagous and predatory invertebrates in mixed forests compared to pure forests during advanced decay stages. This pattern likely stems from the structurally complex and heterogeneous habitats in mixed forests, which provide spatiotemporally continuous and diverse resources. Given that saprophagous invertebrates exhibit lower dependency on tree-specific resources while favoring general detritus and microorganisms, the enhanced microhabitat diversity and sustained nutrient availability in mixed forests offer superior ecological niches for these taxa. In summary, the decomposition process drives a sequential succession of invertebrate functional groups. Rather than remaining static, the invertebrate community undergoes predictable reorganization in response to shifting resource availability and environmental conditions. This functional succession mirrors the transformation of woody debris from a “plant resource” to a “humus resource,” demonstrating their collective integration into a complex food web that synergistically facilitates decomposition processes [47].

We further observed significant differences in invertebrate community composition among distinct stand types within identical decay classes during initial decomposition stages. As decomposition advanced, community structure demonstrated increasing convergence across both stand types and decay classes (Figure 3 and Figure 4), consistent with patterns reported by Zuo et al. [17]. This convergence indicates that, although initial variations in chemical properties and decomposition rates among tree species drove distinct community differentiation across stand types and decay stages during early phases, prolonged decomposition promoted progressive bark shedding and microenvironmental homogenization in xylem tissues across tree species. Concurrently, complex organic macromolecules underwent degradation into simpler, structurally analogous small molecules, substantially reducing substrate heterogeneity. This physicochemical convergence subsequently drove invertebrate community homogenization. Consequently, dynamic shifts in the properties and resource availability of woody debris facilitated tighter coupling of diverse invertebrate assemblages to decomposition processes [48].

PLS-PM analysis revealed that decay stage and tree species superseded stand type as the dominant factors controlling the ecological process (Figure 5). Their significant effects on wood density and TN content confirm that decomposition inherently constitutes a dual process of physical structural disintegration and nutrient enrichment [49]. This physicochemical transition provides a mechanistic foundation for invertebrate community succession. Contrastingly, tree species primarily influenced initial decay stages by establishing substrate baseline properties. Furthermore, decay class and tree species indirectly governed invertebrate diversity and functional group responses through modifications of the physicochemical characteristics of woody debris [50].

Specifically, phytophagous groups experienced indirect suppression via decay class-mediated reductions in wood density and TN content. Deterioration of the physical structure of the wood diminished suitable habitats and resources for specialist feeders (Figure 5). Conversely, saprophagous groups exhibited multi-driven responses. Altered wood density and lignin content provided refuge opportunities while elevated nitrogen availability stimulated microbial proliferation. This microbial activity both directly and indirectly provisioned food resources for saprophagous invertebrates, enhancing their abundance and subsequently sustaining higher trophic levels, including predators.

In summary, the characteristics of woody debris profoundly influence material cycling and invertebrate community diversity. Therefore, retaining a certain amount of woody debris and preserving its full decay sequence in forests is crucial. Particularly in plantation management, emphasis should shift beyond mere wood stock metrics toward safeguarding forest ecological and species diversity. Each decay stage of woody debris sustains a unique combination of functional groups, forming the foundation for maintaining complex soil food webs and ecosystem functions.

## 5. Conclusions

By integrating the effects of tree species, forest type, and decay stage, this study elucidates the ecological mechanisms driving invertebrate community dynamics. The results demonstrate that decay stage and tree species dominate the successional direction of invertebrate communities by directly affecting the wood density and total nitrogen content of woody debris. Their influence overrides the initial effects of tree species (e.g., *Betula platyphylla* and *Larix principisrupprechtii*) and forest type (pure vs. mixed forests) during the middle to late decay stages, leading to a convergence in the community structure of invertebrates across different tree species and forest types. As woody debris transitions from initial to advanced decay stages, phytophagous invertebrate abundance declines significantly, whereas saprophagous invertebrate abundance increases markedly, thereby supporting a rise in predatory invertebrate abundance. Moreover, mixed forest management is critical for biodiversity maintenance. Compared to pure forests, mixed forests provide more diverse woody debris substrates and form more heterogeneous microhabitats, which collectively sustain greater functional group diversity and more complex food webs, thereby enhancing ecosystem stability and resilience.

## Figures and Tables

**Table 1:** The attributes of the sample plots and the sample trees were outlined.

Forest Type	Plot Number	Age (Year)	Longitude E	Altitude N	Density (Tree/hm^2^)	DBH (cm)	Tree Height (m)	Canopy Density
Larch Forest	L1	43a	117°50′	42°47′	447	21.60	13.10	0.65
	L2	46a	117°50′	42°47′	631	25.70	13.80	0.86
	L3	44a	117°53′	42°48′	382	22.10	13.20	0.60
	L4	42a	117°44′	42°43′	462	21.53	17.25	0.63
	L5	42a	117°42′	42°41′	586	20.14	15.85	0.78
	L6	45a	117°06′	42°44′	608	25.89	19.95	0.85
Birch Forest	B1	40a	117°53′	42°49′	448	17.30	11.95	0.73
	B2	42a	117°32′	42°46′	486	24.21	16.30	0.68
	B3	42a	117°38′	42°45′	311	22.68	17.20	0.56
Larch–Birch Mixed Forest	LB1	45a	117°49′	42°47′	426	25.90	14.20	0.61
	LB2	45a	117°49′	42°47′	588	23.80	14.90	0.72
	LB3	40a	117°51′	42°48′	513	16.03	10.30	0.70
	LB4	40a	117°52′	42°48′	469	20.10	15.00	0.80
	LB5	40a	117°53′	42°48′	619	18.73	10.20	0.81
	LB6	42a	117°53′	42°48′	576	20.22	12.55	0.77
	LB7	46a	117°40′	42°42′	533	26.11	21.95	0.82
	LB8	42a	117°41′	42°41′	516	20.94	16.35	0.85
	LB9	40a	117°42′	42°41′	540	18.59	14.10	0.82
	LB10	48a	117°41′	42°44′	467	30.90	16.85	0.77

Note: L1~L6 represent sample plot 1 to 6 in the Larch Forest, B1~B3 represent sample plot 1 to 6 in the Birch Forest, LB1~LB10 represent sample plot 1 to 6 in the Larch–Birch Mixed Forest.

**Table 2:** The physicochemical characteristics of different woody debris.

Decay Class	Mixed Forest		Pure Forest	
	BM	LM	BP	LP
Moisture Content (%)				
I	0.48 ± 0.12 ABa	0.22 ± 0.04 Aa	0.40 ± 0.12 Ba	0.24 ± 0.06 Aa
II	0.59 ± 0.17 Aa	0.37 ± 0.12 Aa	0.65 ± 0.14 Aa	0.29 ± 0.09 Aa
III	0.59 ± 0.14 Aa	0.43 ± 0.19 Aa	0.56 ± 0.16 Aa	0.42 ± 0.08 Aa
IV	0.65 ± 0.17 Aa	0.54 ± 0.16 Aa	0.66 ± 0.17 Aa	0.51 ± 0.18 Aa
V	0.73 ± 0.18 Aa	0.67 ± 0.09 Aa	0.72 ± 0.17 Aa	0.58 ± 0.25 Aa
Density (g/cm^3^)				
I	0.32 ± 0.10 Ab	0.48 ± 0.11 Bc	0.35 ± 0.07 Ab	0.49 ± 0.09 Bb
II	0.24 ± 0.11 Aa	0.43 ± 0.08 Ac	0.17 ± 0.09 Aa	0.45 ± 0.10 Bb
III	0.25 ± 0.12 Aa	0.34 ± 0.12 Ab	0.25 ± 0.12 Aa	0.41 ± 0.03 Bb
IV	0.22 ± 0.08 Aa	0.30 ± 0.11 Ab	0.23 ± 0.14 Aa	0.33 ± 0.11 Ba
V	0.20 ± 0.17 Aa	0.18 ± 0.07 Aa	0.15 ± 0.11 Aa	0.28 ± 0.16 Aa
Total Nitrogen Content (TN) (%)				
I	0.21 ± 0.08 Aa	0.13 ± 0.07 Aa	0.21 ± 0.06 Aa	0.12 ± 0.06 Aa
II	0.42 ± 0.21 Aa	0.13 ± 0.12 Aa	0.43 ± 0.19 Aa	0.13 ± 0.06 Aa
III	0.34 ± 0.14 Aa	0.31 ± 0.16 Aa	0.32 ± 0.09 Aa	0.14 ± 0.06 Aa
IV	0.45 ± 0.10 Aa	0.41 ± 0.22 Aa	0.37 ± 0.15 Aa	0.27 ± 0.16 Aa
V	0.69 ± 0.33 Aa	0.73 ± 0.21 Aa	0.50 ± 0.25 Aa	0.35 ± 0.29 Aa
Total Carbon Content (TC) (%)				
I	47.97 ± 0.46 Aa	48.59 ± 0.77 ABa	47.36 ± 0.94 Aa	48.58 ± 0.73 Ba
II	48.27 ± 0.98 Aa	48.38 ± 1.11 Aa	46.19 ± 3.83 Aa	48.38 ± 0.75 Aa
III	48.45 ± 1.24 Aa	49.18 ± 1.56 Aa	47.51 ± 2.00 Aa	48.68 ± 0.47 Aa
IV	48.03 ± 1.00 Aa	48.58 ± 1.48 Aa	48.09 ± 0.85 Aa	48.74 ± 2.14 Aa
V	47.54 ± 1.17 Aa	48.01 ± 1.22 Aa	47.08 ± 3.26 Aa	49.32 ± 2.12 Aa
C/N				
I	259.45 ± 112.63 Ab	524.32 ± 345.98 Ab	241.19 ± 71.75 Ab	518.60 ± 306.01 Aa
II	133.91 ± 48.46 Aa	561.24 ± 259.09 ABb	127.84 ± 61.94 Aa	448.41 ± 166.21 Ba
III	162.59 ± 69.52 Aa	222.54 ± 158.85 Aa	159.67 ± 46.92 Aa	447.86 ± 257.67 Ba
IV	111.28 ± 21.66 Aa	153.02 ± 82.74 Aa	150.68 ± 72.20 Aa	221.85 ± 84.99 Ba
V	84.25 ± 40.19 Aa	71.61 ± 23.19 Aa	126.60 ± 83.10 Aa	298.03 ± 238.65 Ba
Lignin Content (%)				
I	33.27 ± 9.84 Aa	23.07 ± 6.18 Aa	27.40 ± 7.96 Aa	25.54 ± 4.13 Aa
II	33.41 ± 5.41 Aa	22.19 ± 7.60 Aa	27.04 ± 6.07 Aa	26.28 ± 5.22 Aa
III	29.51 ± 8.25 Aa	29.15 ± 8.78 Aa	25.178 ± 7.06 Aa	21.53 ± 3.44 Aa
IV	31.86 ± 10.32 Aa	27.31 ± 3.08 Aa	29.66 ± 3.52 Aa	25.51 ± 8.25 Aa
V	31.47 ± 4.89 Aa	30.51 ± 6.71 Aa	26.44 ± 6.26 Aa	27.63 ± 8.50 Aa
Cellulose Content (%)				
I	35.18 ± 3.57 Ab	32.6.75 ± 5.63 Aa	32.02 ± 9.20 Aa	31.13 ± 6.83 Aa
II	30.87 ± 5.99 Aa	32.2.73 ± 8.71 Aa	28.78 ± 10.86 Aa	36.71 ± 4.23 Aa
III	31.14 ± 2.96 Aa	30.4.39 ± 3.54 Aa	36.29 ± 7.70 Aa	35.12 ± 8.87 Aa
IV	30.07 ± 8.44 Aa	31.5.10 ± 8.48 Aa	32.06 ± 11.19 Aa	29.83 ± 5.68 Aa
V	21.65 ± 10.06 Aa	25.0.44 ± 5.93 Aa	28.83 ± 12.27 Aa	25.54 ± 12.42 Aa

Note: Values are presented as mean ± SE. Capital letters indicate differences between woody debris types across different forest types under the same treatment, while lowercase letters indicate differences between woody debris types across different decomposition levels under the same treatment (*p* < 0.05). I–V denotes the physicochemical properties of woody debris. LP refers to *Larix principis-rupprechtii* woody debris in pure forests, LM refers to *L. principis-rupprechtii* woody debris in mixed forests, BP refers to *B. platyphylla* woody debris in pure forests, and BM refers to *B. platyphylla* woody debris in mixed forests; the same notations are used below.

**Table 3:** Invertebrate abundance in woody debris by phylum, class, and order.

Taxonomic Group	Number of Individuals	Fraction (%)
All samples	4312	100
Annelida	49	1.14
Oligochaeta	49	1.14
Arthropoda	4263	98.86
Arachnida	380	8.81
Araneae	180	4.17
Pseudoscorpiones	27	0.63
Opiliones	173	4.01
Diplopoda	31	0.72
Julida	31	0.72
Chilopoda	572	13.27
Scolopendromorpha	557	12.92
Geophilomorpha	12	0.28
Scutigeromorpha	3	0.07
Insecta	3280	76.06
Hemiptera	46	1.07
Psocoptera	6	0.14
Thysanoptera	82	1.90
Coleoptera	1355	31.42
Neuroptera	7	0.16
Lepidoptera	14	0.32
Diptera	1739	40.33
Hymenoptera	31	0.72

**Table 4:** Multivariate analysis of variance of the effects of tree species, forest type, and decay class on invertebrate diversity.

	df	Shannon–Wiener Index	Pielou’s Index	Individual Density	Group Number
Tree Species	1	1.531	0.904	8.792 **	1.642
Forest Types	1	7.407 **	3.06	0.08	4.726 *
Decay class	4	1.421	1.143	25.415 ***	4.689 **
Tree Species × Forest Types	1	0.418	0.051	0.689	0.553
Tree Species × Decay Class	4	2.883 *	1.084	1.205	2.868 *
Forest Types × Decay Class	4	1.516	1.494	1.991	0.520
Tree Species × Forest Types × Decay class	4	1.384	0.927	1.341	1.187

Note: *p* < 0.05: *; *p* < 0.01: **; *p* < 0.001: ***.

## Data Availability

Data are available upon request to the corresponding authors.

## Supplementary Materials

The following supporting information can be downloaded at: https://www.mdpi.com/article/10.3390/biology15010043/s1, Figure S1: Diversity of macroinvertebrates in different woody debris; Table S1: Invertebrate community composition in different types of woody debris.

## Appendix A

**Table A1:** The impact of woody debris on soil nitrogen and carbon content.

Decay Class	Mixed Forest		Pure Forest	
	BM	LM	BP	BM
Soil TN(%)				
I	0.76 ± 0.29 Aa	0.44 ± 0.16 Bb	0.35 ± 0.12 Ba	0.37 ± 0.11 Ba
II	0.57 ± 0.31 Ab	0.49 ± 0.21 Ab	0.45 ± 0.13 Aa	0.48 ± 0.26 Aa
III	0.62 ± 0.33 Ab	0.76 ± 0.48 Aa	0.39 ± 0.06 Aa	0.36 ± 0.18 Ab
IV	0.67 ± 0.28 Bb	0.82 ± 0.13 Ba	0.34 ± 0.07 Ba	0.47 ± 0.19 Bb
V	0.99 ± 0.24 Aa	0.89 ± 0.21 Aa	0.69 ± 0.44 Ab	0.71 ± 0.42 Ab
Soil TN (CK)	0.80 ± 0.29 Ba	0.80 ± 0.29 Ba	0.48 ± 0.17 Aa	0.57 ± 0.28 Ab
Soil TC(%)				
I	11.80 ± 3.28 Ba	8.61 ± 4.93 Aa	4.38 ± 1.61 Aa	7.81 ± 7.15 Aa
II	11.06 ± 4.60 Ba	10.39 ± 5.10 Ba	5.94 ± 1.97 Aa	6.47 ± 3.69 Aa
III	8.48 ± 4.89 Aa	11.64 ± 7.90 Aa	5.29 ± 1.54 Aa	4.66 ± 3.10 Aa
IV	9.96 ± 4.36 Ba	12.38 ± 1.87 Ba	4.11 ± 0.86 Ba	6.94 ± 3.78 Ba
V	11.14 ± 4.01 Ba	13.77 ± 3.93 Ba	4.56 ± 1.63 Ba	6.88 ± 3.63 Ba
Soil TC (CK)	11.84 ± 4.58 Ba	11.84 ± 4.58 Ba	6.75 ± 2.36 A	6.67 ± 2.53 Ba
Soil C/N				
I	19.38 ± 15.74 Aa	21.37 ± 16.99 Aa	12.31 ± 0.60 Aa	22.97 ± 25.78 Aa
II	25.07 ± 19.65 Ab	24.54 ± 19.66 Aa	12.93 ± 1.02 Aa	13.19 ± 0.92 Ab
III	13.20 ± 2.06 Aa	14.93 ± 1.51 Aa	13.36 ± 3.07 Aa	12.36 ± 1.72 Ab
IV	14.89 ± 1.46 Aa	15.16 ± 1.46 Aa	12.10 ± 0.68 Ba	14.01 ± 1.86 Ab
V	11.88 ± 4.34 Aa	15.39 ± 0.87 Aa	8.93 ± 4.57 Bb	10.98 ± 3.98 Ab
Soil C/N (CK)	14.67 ± 1.34 Aa	14.67 ± 1.34 Aa	13.93 ± 1.73 Aa	12.51 ± 3.31 Ab

Note: Values are presented as mean ± SE. Capital letters indicate differences between woody debris types across different forest types under the same treatment, while lowercase letters indicate differences between woody debris types across different decomposition levels under the same treatment (*p* < 0.05). LP refers to the woody debris of *L. principis-rupprechtii* Pure; LM refers to the woody debris of *L. principis-rupprechtii* Mixed; BP refers to the woody debris of *B. platyphylla* Pure; and BM refers to the woody debris of *B. platyphylla* Mixed. I-V denotes the soil nutrients under woody debris. CK denotes the soil nutrient content in the absence of woody debris coverage.

## References

[1] Sverdrup-Thygeson A., Ims R.A. (2002). The effect of forest clearcutting in Norway on the community of saproxylic beetles on aspen. Biol. Conserv..

[2] Cornelissen J.H., Sass-Klaassen U., Poorter L., Geffen K., Logtestijn R.S., Hal J., Goudzwaard L., Sterck F.J., Klaassen R.K., Freschet G.T. (2012). Controls on coarse wood decay in temperate tree species: Birth of the LOGLIFE experiment. Ambio.

[3] Harmon M., Franklin J., Swanson F., Sollins P., Gregory S., Lattin J., Anderson N., Cline S., Aumen N., Sedell J. (2004). Ecology of coarse woody debris in temperate ecosystems. Adv. Ecol. Res..

[4] Seibold S., Rammer W., Hothorn T., Seidl R., Ulyshen M.D., Lorz J., Cadotte M.W., Lindenmayer D.B., Adhikari Y.P., Aragón R. (2021). The contribution of insects to global forest deadwood decomposition. Nature.

[5] Ulyshen M.D. (2016). Wood decomposition as influenced by invertebrates. Biol. Rev. Camb. Philos. Soc..

[6] Jean-François D., Ira H. (2010). The ecology of saprophagous macroarthropods (millipedes, woodlice) in the context of global change. Biol. Rev. Camb. Philos. Soc..

[7] Taylor M.K., Ulyshen M.D., Horn S., Poole E.M., Callaham M.A. (2024). Variation in the contribution of macroinvertebrates to wood decomposition as it progresses. Ecosphere.

[8] Zuo J., Fonck M., Hal J., Cornelissen J.H.C., Berg M.P. (2014). Diversity of macro-detritivores in dead wood is influenced by tree species, decay stage and environment. Soil Biol. Biochem..

[9] Seibold S., Wolfgang W.W., Didem A., Martin M.G., Akira M., Marc W.C., Jonas H., Claus B., Simon T. (2023). Drivers of community assembly change during succession in wood-decomposing, cbeetle communities. Anim. Ecol..

[10] Fujii S., Takeda H. (2017). Succession of soil microarthropod communities during the aboveground and belowground litter decomposition processes. Soil Biol. Biochem..

[11] Kamczyc J., Dyderski M.K., Horodecki P., Jagodzinski A.M. (2019). Mite Communities (Acari, Mesostigmata) in the Initially Decomposed “Litter Islands” of 11 Tree Species in Scots Pine (*Pinus sylvestris* L.) Forest. Forests.

[12] Guo C., Tuo B., Ci H., Yan E.R., Cornelissen J.H. (2021). Dynamic Feedbacks among Tree Functional Traits, Termite Populations and Deadwood Turnover. J. Ecol..

[13] Zuo J., Cornelissen J.H., Hefting M.M., Sass-Klaassen U., Richard S.P., Jurgen H., Goudzwaard L., Liu J.C., Berg M.P. (2016). The (w)hole story: Facilitation of dead wood fauna by bark beetles?. Soil Biol. Biochem..

[14] Ulyshen M.D., Hanula J.L. (2010). Patterns of saproxylic beetle succession in loblolly pine. Agric. For. Entomol..

[15] Magnússon R.Í., Tietema A., Cornelissen J.H., Hefting M.M., Kalbitz K. (2016). Tamm Review: Sequestration of carbon from coarse woody debris in forest soils. For. Ecol. Manag..

[16] Lasota J., Błońska E., Piaszczyk W., Wiecheć M. (2018). How the deadwood of different tree species in various stages of decomposition affected nutrient dynamics?. J. Soils Sediments.

[17] Zuo J., Berg M.P., Jurgen H., Richard S.P., Leo G., Hefting M., Lourens P., Frank J.S., Cornelissen J.H. (2021). Fauna community convergence during decomposition of deadwood across tree species and forests. Ecosystems.

[18] Natalie C. (2023). Let sleeping logs lie: Beta diversity increases in deadwood beetle communities over time. J. Anim. Ecol..

[19] Brassard W.B., Chen H.Y. (2008). Effects of Forest Type and Disturbance on Diversity of Coarse Woody Debris in Boreal Forest. Ecosystems.

[20] Zuo J., Muys B., Berg M.P., Hefting M.M., Richard S.P., Jurgen H., Cornelissen J.H. (2023). Earthworms are not just “earth” worms: Multiple drivers to large diversity in Deadwood. For. Ecol. Manag..

[21] Lu C.T., Li Y., Xiao J.J., Dai Z.L. (2013). Characteristics of soil fauna community of three plantations in the western Sichuan Basin border of China. Chin. J. Appl. Environ. Biol..

[22] Plath E., Fischer K. (2024). Spruce dieback as chance for biodiversity: Standing deadwood promotes beetle diversity in post-disturbance stands in western Germany. J. Insect Conserv..

[23] Felix N., Jonas H., Rafael A., Didem A., Christian A., Peter S., Sebastian S., Michael S., Wolfgang W.W., Martin M.G. (2022). Hierarchical trait filtering at different spatial scales determines beetle assemblages in deadwood. Funct. Ecol..

[24] Piętka S., Sotnik A., Damszel M., Sierota Z. (2019). Coarse woody debris and wood-colonizing fungi differences between a reserve stand and a managed forest in the Taborz region of Poland. J. For. Res..

[25] Andreas F., Dirk K., Tobias M., Marcus D., Renate R., Björn H., Eduard L.K. (2015). Diversity and Interactions of Wood-Inhabiting Fungi and Beetles after Deadwood Enrichment. PLoS ONE.

[26] Oettel J., Zolles A., Gschwantner T., Kindermann G., Manfred K.S., Gossner M.M., Essl F. (2023). Dynamics of standing deadwood in Austrian forests under varying forest management and climatic conditions. J. Appl. Ecol..

[27] Spies T.A., Franklin J.F., Thomas T.B. (1988). Coarse woody debris in douglas-fir forests of western oregon and Washington. Ecology.

[28] Rouvinen S., Kuuluvainen T., Karjalainen L. (2002). Coarse woody debris in old Pinus sylvestris dominated forests along a geographic and human impact gradient in boreal Fennoscandia. Can. J. For. Res..

[29] Harmon M.E., Sexton J. (1996). Guidelines for Measurements of Woody Detritus in Forest Ecosystems.

[30] Yin W.Y. (1998). Pictorical Key to Soil Animals of China.

[31] Yang G.R., Dou P.P., Ma Y., Wang H.J., Lin D.M. (2020). Characteristics and influencing factors of surface soil fauna community in a subtropical evergreen broad-leaved forest of Jinfo Mountain. Acta Ecol. Sin..

[32] Zhang A.J., Zhang J., Li J.J., Liu Z.G., Zhang D.J. (2020). Characteristics of soil faunal community structure before and after the rotation period of Eucalyptus grandis plantations with various densities. Acta Ecol. Sin..

[33] Ouyang S., Xiang W., Gou M., Chen L., Lei P., Xiao W., Deng X., Zeng L., Li J., Zhang T. (2020). Stability in subtropical forests: The role of tree species diversity, stand structure, environmental and socio-economic conditions. Glob. Ecol. Biogeogr..

[34] Xie J., Yan Q., Yuan J., Li R., Lü X., Liu S., Zhu J. (2020). Temporal Effects of Thinning on the Leaf C:N:P Stoichiometry of Regenerated Broadleaved Trees in Larch Plantations. Forests.

[35] Hafner S.D., Groffman P.M., Mitchell M.J. (2005). Leaching of dissolved organic carbon, dissolved organic nitrogen, and other solutes from coarse woody debris and litter in a mixed forest in New York State. Biogeochemistry.

[36] Lajtha K., Crow S.E., Yano Y., Kaushal S.S., Sulzman E., Sollins P., Spears J.D. (2005). Detrital controls on soil solution N and dissolved organic matter in soils: A field experiment. Biogeochemistry.

[37] Tan B., Yin R., Zhang J., Xu Z.F., Liu Y., He S.Q., Zhang L., Li H., Wang L.X., Liu S.N. (2020). Temperature and moisture modulate the contribution of soil fauna to litter decomposition via different pathways. Ecosystems.

[38] Paletto A., Tosi V. (2010). Deadwood density variation with decay class in seven tree speciesofthe ltalian Alps. Scand. J. For. Res..

[39] Pastorelli R., Paletto A., Agnelli A.E., Lagomarsino A., Meo I.D. (2020). Microbial communities associated with decomposing deadwood of downy birch in a natural forest in Khibiny Mountains (Kola Peninsula, Russian Federation). For. Ecol. Manag..

[40] Chen L.X., Xiang W.H., Wu H.L., Lei P.F., Zhang S.L., Ouyang S., Deng X.W., Fang X. (2017). Tree growth traits and social status affect the wood density of pioneer species in secondary subtropical forest. Ecol Evol.

[41] Dossa G.G., Schaefer D., Zhang J.L., Tao J.P., Cao K.F., Corlett R.T., Cunningham A.B., Xu J.C., Cornelissen J.H., Harrison R.D. (2018). The cover uncovered: Bark control over wood decomposition. J. Ecol..

[42] Zuo J., Hefting M.M., Berg M.P., Richard S.P., Hal J., Goudzwaard L., Liu J.C., Sass-klaassen U., Sterck F.J., Poorter L. (2018). Is there a tree economics spectrum of decomposability?. Soil Biol. Biochem..

[43] Müller J., Wende B., Strobl C., Eugster M., Gallenberger I., Floren A., Steffan-Dewenter I., Linsenmair K.E., Weisser W.W., Gossner M.M. (2015). Forest management and regional tree composition drive the host preference of saproxylic beetle communities. J. Appl. Ecol..

[44] Saint-Germain M., Drapeau P., Buddle M.C. (2007). Host-use patterns of saproxylic phloeophagous and xylophagous coleoptera adults and larvae along the decay gradient in standing dead black spruce and aspen. Ecography.

[45] Tian H.X., Cheng X.Q., Han H.R., Jing H.Y., Liu X.J., Li Z.Z. (2019). Seasonal variations and thinning effects on soil phosphorus fractions in *Larix principis-rupprechtii* Mayr. Plantations. Forests.

[46] Vanderwel C.M., Malcolm R.J., Smith M.S. (2006). An integrated model for snag and downed woody debris decay class transitions. For. Ecol. Manag..

[47] Parisi F., Pioli S., Lombardi F., Fravolini G., Marchetti M., Tognetti R. (2018). Linking deadwood traits with saproxylic invertebrates and fungi in European forests—A review. iForest-Biogeosci. For..

[48] Laureline L., Irene C., Camille M., Yoan P., Wilfried T., Lucie V., Georges K. (2023). Beyond the role of climate and soil conditions: Living and dead trees matter for soil biodiversity in mountain Forests. Soil Biol. Biochem..

[49] Jonsell M., Hansson J., Wedmo L. (2007). Diversity of saproxylic beetle species in logging residues in Sweden—Comparisons between tree species and diameters. Biol. Conserv..

[50] Andringa J.I., Zuo J., Berg M.P., Klein R., Veer J., Geus R., Beaumont M., Goudzwaard L., Hal J., Broekman R. (2019). Combining tree species and decay stages to increase invertebrate diversity in dead wood. For. Ecol. Manag..

