# Supplementary material for: Invertebrate Communities and Driving Factors Across Woody Debris Types in Temperate Forests, Northern China
Source: Biology (Basel). 2025 Dec 26;15(1):43. doi: 10.3390/biology15010043 (PMC12784839; doi:10.3390/biology15010043)
Supplement: Supplementary file 1 [file biology-15-00043-s001.zip › Figure S1.pdf]

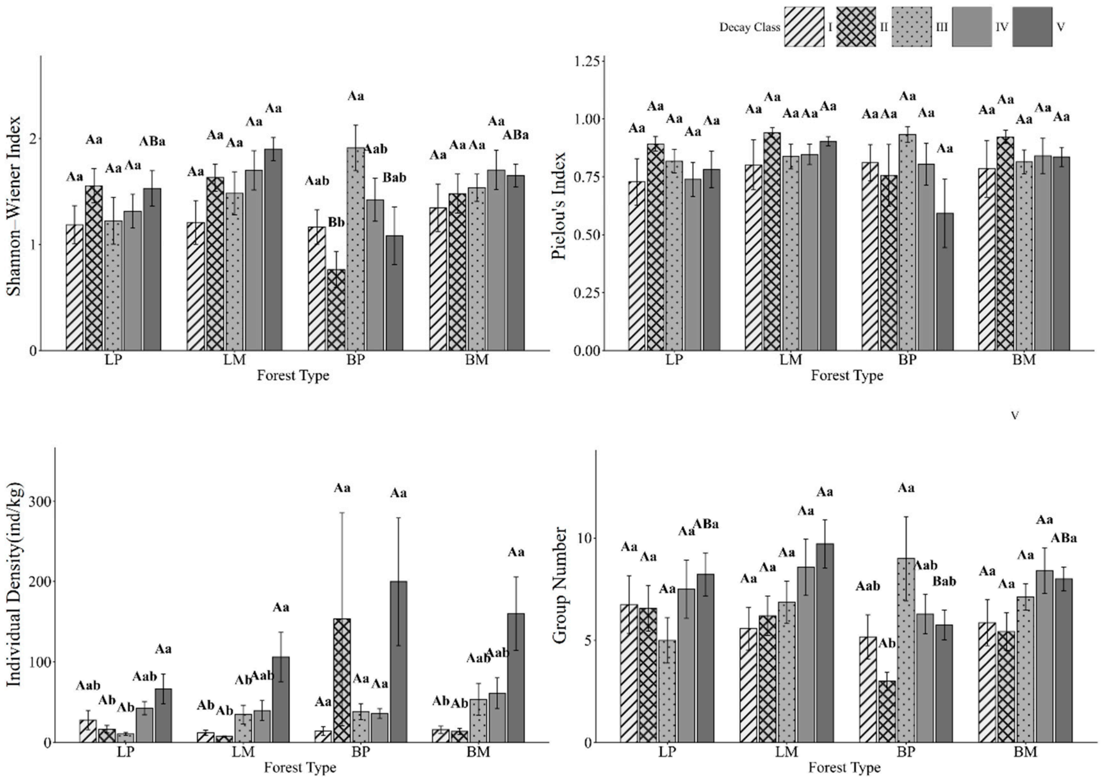

Figure S1. Values are presented as mean ± SE. Differences in macroinvertebrate diversity were tested for significance using one-way analysis of variance (ANOVA). Diversity of macroinvertebrates in different woody debris: Capital letters indicate significant differences between woody debris types across different forest types under the same treatment, while lowercase letters indicate significant differences between woody debris types across different decomposition levels under the same treatment ($p < 0.05$).
