# Supplementary material for: Invertebrate Communities and Driving Factors Across Woody Debris Types in Temperate Forests, Northern China
Source: Biology (Basel). 2025 Dec 26;15(1):43. doi: 10.3390/biology15010043 (PMC12784839; doi:10.3390/biology15010043)
Supplement: Supplementary file 1 [file biology-15-00043-s001.zip › Table S1.pdf]

Table S1. Invertebrate community composition in different types of woody debris.

					**Mixed Forest**										**Pure Forest**					
**Family**			**BM**					**LM**					**BP**							
	**I**	**II**	**III**	**IV**	**V**	**I**	**II**	**III**	**IV**	**V**	**I**	**II**	**III**	**IV**	**V**	**I**	**II**	**III**	**IV**	**V**
Tubificidae	0±0Aa	0.09±0.23Aa	1.57±4.43Aa	0±0Ba	2.38±5.74Aa	0±0Aa	0±0Aa	0±0Aa	3.53±6.02Aa	0.43±1.13Aa	0.36±0.88Aa	0±0Aa	1.73±2.36Aa	1.64±3.64ABa	1.49±3.72Aa	0±0Aa	0±0Aa	0±0Aa	0±0Ba	0±0Aa
Enchytraeidae	0±0Aa	0±0Aa	0±0Aa	0±0Aa	1.39±4.61Aa	0±0Aa	0.21±0.47Aa	0±0Aa	0±0Aa	0±0Aa	0±0Aa	0±0Aa	0±0Aa	1.33±2.82Aa	1.33±3.76Aa	0±0Aa	0±0Aa	0±0Aa	0±0Aa	0.1±0.29Aa
Lithobiidae	0.99±1.1Ab	2.42±2.54ab	6.89±8.2ABab	10.99±11.81Aa	27.38±30.86Aa	2.11±3.14Ab	1.08±1.19Ab	7.62±6.57Aab	16.51±17.21Aab	35.15±38.93Aa	1.79±1.76Aa	7.54±8.09Aa	5.82±2.6Aa	5.11±6.62Aa	16.89±22.1Aa	0.67±0.77Ab	2.59±4.33Aab	1.83±1.73Bab	10.45±10.65Aa	16.48±25.14Aa
Scutigeridae	0±0Aa	0±0Aa	0±0Aa	0±0Aa	0±0Aa	0±0Aa	0±0Aa	0±0Aa	0±0Aa	0.77±2.05Aa	0±0Aa	0±0Aa	0±0Aa	0±0Aa	0±0Aa	0.02±0.05Aa	0±0Aa	0±0Aa	0±0Aa	0±0Aa
Schendy lidae	0±0Aa	0±0Aa	0±0Aa	0.07±0.24Aa	0±0Aa	0±0Aa	0.21±0.47Aa	0±0Aa	0.05±0.13Aa	0±0Aa	0±0Aa	0±0Aa	0±0Aa	0±0Aa	0.89±2.53Aa	0±0Aa	0±0Aa	0±0Aa	0.19±0.35Aa	1.31±3.93Aa
Mongolulidae	0±0Aa	0±0Aa	0±0Aa	0.07±0.24Aa	0±0Aa	0±0Aa	0±0Aa	0±0Aa	0±0Aa	1.04±2.76Aa	0.26±0.64Aa	0±0Aa	0±0Aa	0±0Aa	0±0Aa	0±0Aa	0±0Aa	0±0Aa	0.1±0.29Aa	0±0Aa
Julidae	0.08±0.22Aa	0±0Aa	0±0Aa	0.51±1.6Aa	1.93±5.16Aa	0±0Aa	0.51±0.72Aa	0±0Aa	1.77±2.86Aa	1.42±1.88Aa	0±0Aa	0±0Aa	0±0Aa	0.21±0.56Aa	0±0Aa	0±0Aa	0.17±0.46Aa	0±0Aa	0±0Aa	0.44±1.31Aa
Phalangiidae	1.37±1.53Aa	0.67±1.41Ba	2.05±3.28A	2.99±3.53Aa	4.31±6.69Aa	1.96±1.38Aa	0±0Ba	3.04±4.04Aa	1.19±1.52Aa	4.12±6.33ABa	0.27±0.44Aa	0±0Ba	0±0Ba	0.25±0.66Aa	0±0Ba	2.51±2.71Aa	3.68±2.73Aa	0±0Bc	1.18±1.62Aabc	0.47±1.16ABbc
Gnaphosidae	0±0Aa	0±0Aa	0±0Aa	0.19±0.6Aa	0.16±0.53Aa	0±0Aa	0.21±0.47Aa	0±0Aa	0±0Aa	0±0Aa	0±0Aa	0±0Aa	0.11±0.24Aa	0±0Aa	3.57±10.1Aa	0.07±0.21Aa	0.18±0.48Aa	0.17±0.46Aa	0.2±0.37Aa	0±0Aa
Agelenidae	0.16±0.43Aa	0.18±0.48Aa	0.17±0.48Aa	0.17±0.55Aa	20.14±66.79Aa	0.19±0.5Aa	0±0Aa	0±0Aa	0.35±0.65Aa	0.35±0.92Aa	0±0Aa	0±0Aa	0±0Aa	0±0Aa	0±0Aa	0.15±0.31Aa	0±0Aa	0±0Aa	0.42±1.2Aa	0.1±0.31Aa
Lycosidae	0±0Aa	0±0Aa	0±0Aa	0.44±0.95Aa	0±0Aa	0.05±0.14Aa	0±0Aa	0.66±1.36Aa	0.24±0.63Aa	0.38±1.01Aa	0±0Aa	4.13±7.27Aa	0.21±0.48Aa	0.24±0.41Aa	2.96±7.44Aa	0.05±0.15Aa	0.54±1.19Aa	0±0Aa	0±0Aa	0.05±0.15Aa
Linyphiidae	0±0Aa	0±0Aa	0.34±0.96Aa	0±0Aa	0±0Aa	0±0Aa	0±0Aa	0±0Aa	0.35±0.65Aa	0.35±0.92Aa	0.13±0.32Aa	1.49±3.64Aa	0±0Aa	0±0Aa	0±0Aa	0.11±0.3Aa	0±0Aa	0±0Aa	0±0Aa	0±0Aa
Araneidae	0±0Aa	0±0Aa	0±0Aa	0±0Aa	0.32±0.73Aa	0±0Aa	0±0Aa	0±0Aa	0±0Aa	2.54±5.71Aa	0±0Aa	0±0Aa	0±0Aa	0±0Aa	0±0Aa	0.05±0.15Aa	0±0Aa	0.06±0.17Aa	0±0Aa	0±0Aa
Thomisidae	0.17±0.45Aa	0±0Aa	0±0Aa	0±0Aa	0±0Aa	0±0Aa	0±0Aa	0±0Aa	0±0Aa	0±0Aa	0.09±0.22Aa	0±0Aa	0.33±0.75Aa	0±0Aa	0±0Aa	0.24±0.67Aa	0.23±0.6Aa	0±0Aa	0±0Aa	0.1±0.29Aa
Anyphaenidae	0.7±1.27Aa	0.09±0.23Aa	0±0Aa	0±0Aa	0±0Aa	0.09±0.25Aa	0±0Aa	0±0Aa	0±0Aa	0±0Aa	0±0Aa	0±0Aa	0±0Aa	0±0Aa	1.46±4.14Aa	0±0Aa	0.21±0.55Aa	0±0Aa	0±0Aa	0±0Aa
Oonopidae	0±0Aa	0±0Aa	0±0Aa	0±0Aa	0±0Aa	0.03±0.08Aa	0.07±0.15Aa	0±0Aa	0±0Aa	0±0Aa	0±0Aa	0±0Aa	0±0Aa	0.47±1.25Aa	0±0Aa	0.28±0.67Aa	0±0Aa	0±0Aa	0±0Aa	0.13±0.38Aa
Clubionidae	0.48±1.28Aa	0±0Aa	0±0Aa	0±0Aa	0.42±1.28Aa	0±0Aa	0±0Aa	0.21±0.56Aa	0±0Aa	0.39±1.03Aa	0.85±2.07Aa	0±0Aa	0±0Aa	0±0Aa	0.32±0.92Aa	0±0Aa	0±0Aa	0±0Aa	0±0Aa	0.2±0.59Aa
Liocranidae	0±0Aa	1.77±4.67Aa	0±0Aa	0±0Aa	0±0Aa	0±0Aa	0±0Aa	0±0Aa	0.87±2.14Aa	1.55±4.1Aa	0±0Aa	0±0Aa	0±0Aa	0±0Aa	0±0Aa	0±0Aa	0±0Aa	0±0Aa	0±0Aa	0±0Aa
Theridiidae	0±0Aa	0±0Aa	0.13±0.37Aa	0±0Aa	0.28±0.93Aa	0±0Aa	0±0Aa	0.21±0.37Aa	0.05±0.13Aa	5.18±11.42Aa	0.12±0.29Aa	1.65±4.05Aa	8.29±18.08Aa	0.11±0.28Aa	0±0Aa	0.05±0.15Aa	0.78±1.82Aa	0±0Aa	0.25±0.49Aa	0.54±1.31Aa
Theraphosidae	0±0Aa	0±0Aa	0±0Aa	0±0Aa	0±0Aa	0±0Aa	0.21±0.47Aa	0±0Aa	0.05±0.13Aa	0±0Aa	0±0Aa	0±0Aa	0±0Aa	0±0Aa	0±0Aa	0±0Aa	0±0Aa	0±0Aa	0±0Aa	0±0Aa
Cheliferidae	0±0Aa	0±0Aa	0.97±2.75Aa	0.25±0.8Aa	0±0Ba	0±0Aa	0.09±0.2Aa	0.21±0.38Aa	0.05±0.13Aa	2.28±3.22Aa	0.09±0.22Aa	0±0Aa	0±0Aa	0.42±1.12Aa	0±0Ba	0±0Aa	0.17±0.46Aa	0.13±0.33Aa	0.1±0.29Aa	0.82±1.63ABa
Psocidae	0±0Aa	0±0Aa	0±0Aa	0.52±1.66Aa	0±0Aa	0±0Aa	0±0Aa	0±0Aa	0±0Aa	0±0Aa	0±0Aa	0±0Aa	1.63±3.63Aa	0.06±0.17Aa	0±0Aa	0±0Aa	0±0Aa	0±0Aa	0.16±0.46Aa	0±0Aa
Phlaeothripidae	0.16±0.43Aa	0±0Ba	0.06±0.17Aa	0.19±0.6Aa	0.59±1.96Aa	1.77±3.75Aa	0.33±0.74ABa	2.72±7.2Aa	0.24±0.63Aa	0±0Aa	0±0Aa	0±0Ba	0±0Aa	0±0Aa	0.07±0.21Aa	0±0Aa	4.3±8.79Aa	0.25±0.66Aa	0.31±0.89Aa	0.78±2.35Aa
Reduviidae	0±0Aa	0±0Aa	0±0Aa	0±0Aa	0±0Aa	0±0Aa	0±0Aa	0.08±0.2Aa	0±0Aa	0±0Aa	0±0Aa	0±0Aa	0±0Aa	0±0Aa	0±0Aa	0.02±0.05Aa	0.17±0.46Aa	0±0Aa	0±0Aa	0±0Aa
Lygaeidae	0.24±0.64Aa	0.18±0.48AB	0±0Aa	0±0Aa	4.28±10.29Aa	0.13±0.17Aa	0±0Ba	0.21±0.56Aa	0±0Aa	0±0Aa	0±0Aa	0±0Ba	1.91±3.81Aa	0.06±0.17Aa	0.71±2Aa	0.39±0.75Aa	1.32±1.12Aa	0±0Aa	0.3±0.61Aa	3.96±10.62Aa
Chrysopidae	0±0Aa	0±0Aa	0±0Aa	0.61±1.92Aa	0±0Aa	0±0Aa	0±0Aa	0±0Aa	0±0Aa	0±0Aa	0±0Aa	0±0Aa	0±0Aa	0.06±0.17Aa	0±0Aa	0±0Aa	0±0Aa	0±0Aa	0±0Aa	0±0Aa
Siricidae	0±0Aa	0±0Aa	0±0Aa	0±0Aa	0±0Aa	0.08±0.15Aa	0±0Aa	0±0Aa	0±0Aa	0±0Aa	0±0Aa	0±0Aa	0±0Aa	0±0Aa	0±0Aa	0±0Aa	0.08±0.22Aa	1.84±4.87Aa	0±0Aa	0.49±1.47Aa

Formicidae	0±0Aa	0±0Aa	0.97±2.75Aa	0±0Aa	0±0Aa	0±0Aa	0±0Aa	0.08±0.2Aa	0±0Aa	0±0Aa	0±0Aa	0±0Aa	0±0Aa	0±0Aa	0±0Aa	0.13±0.37Aa	0.17±0.46Aa	0.63±1.67Aa	0±0Aa	0±0Aa
Tipulidae	0.05±0.12Aa	1.15±2.32Aa	2.21±3.97Aa	1.47±1.94	11.52±24.59Aa	0±0Ab	0.76±0.93Aab	0.99±1.7Aab	1.97±2.93Aab	4.09±3.65Aa	0±0Aa	0.76±1.86Aa	1.67±2.07Aa	4.83±12.78Aa	2.93±6.12Aa	0.05±0.15Aa	0±0Aa	0.16±0.29Aa	0±0Aa	1.16±2.34Aa
Chloropidae	5.31±13.51Aa	0±0Aa	0.78±2.21Aa	0.3±0.95	0.43±1.43Aa	0±0Aa	0±0Aa	0±0Aa	0±0Aa	8.75±23.16Aa	0±0Aa	0±0Aa	0±0Aa	0±0Aa	7.45±21.08Aa	0±0Aa	0±0Aa	0±0Aa	0±0Aa	0±0Aa
Tephritidae	0.59±1.55Aa	0±0Aa	0±0Aa	0±0Aa	0±0Aa	0±0Aa	0±0Aa	0±0Aa	0±0Aa	0±0Aa	0.36±0.89Aa	0±0Aa	0±0Aa	0±0Aa	0±0Aa	0±0Aa	0.23±0.6Aa	0±0Aa	0.16±0.46Aa	0±0Aa
Coenomyiidae	0±0Aa	0±0Aa	0.97±2.75Aa	0±0Aa	0.86±2.85Aa	0±0Aa	0±0Aa	0±0Aa	0±0Aa	0±0Aa	0±0Aa	0±0Aa	0±0Aa	0±0Aa	30.34±85.81Aa	0±0Aa	0±0Aa	0±0Aa	0±0Aa	0±0Aa
Bibionidae	0±0Aa	0.26±0.69Aa	0±0Aa	0±0Aa	3.25±10.22Aa	0±0Aa	0.26±0.59Aa	0±0Aa	0±0Aa	0.43±1.13Aa	0±0Aa	0.11±0.28Aa	0±0Aa	0±0Aa	0±0Aa	0±0Aa	0±0Aa	0.37±0.68Aa	0±0Aa	8.67±18.35Aa
Cecidomyiidae	0±0Aa	0±0Aa	1.56±4.42Aa	0±0Aa	0.43±1.43Aa	0±0Aa	0.13±0.3Aa	0±0Aa	0.63±1.26Aa	0±0Aa	0±0Aa	130.71±320.17Aa	0±0Aa	2.66±7.03Aa	0±0Aa	0±0Aa	0±0Aa	0.05±0.13Aa	0±0Aa	0.56±1.17Aa
Psychodidae	0±0Aa	0±0Aa	0±0Aa	0±0Aa	0.19±0.62Aa	0±0Aa	0±0Aa	0±0Aa	0±0Aa	0±0Aa	0±0Aa	0±0Aa	0±0Aa	0±0Aa	0±0Aa	0±0Aa	0±0Aa	0±0Aa	0±0Aa	0±0Aa
Lonchaeidae	0±0Aa	0±0Aa	0.78±1.45Aa	0±0Aa	0±0Aa	0±0Aa	0±0Aa	0±0Aa	0±0Aa	2.32±6.15Aa	0±0Aa	3.47±8.5Aa	0±0Aa	0.74±1.97Aa	10.3±20.39Aa	0±0Aa	0±0Aa	0±0Aa	0±0Aa	1.03±2.45Aa
Dolichopodidae	0±0Aa	0±0Aa	0.17±0.48Aa	4.05±12.81Aa	0.06±0.21Aa	0±0Aa	0±0Aa	0±0Aa	0.11±0.3Aa	0±0Aa	0±0Aa	0±0Aa	0.43±0.96Aa	4.23±8.52Aa	0.3±0.86Aa	0±0Aa	0±0Aa	0±0Aa	0±0Aa	0±0Aa
Culicidae	0±0Aa	0±0Aa	0±0Aa	0.17±0.55Aa	0±0Aa	0±0Aa	0±0Aa	0±0Aa	0±0Aa	0±0Aa	0±0Aa	0±0Aa	0.11±0.24Aa	0±0Aa	0±0Aa	0±0Aa	0±0Aa	0±0Aa	0.1±0.29Aa	0±0Aa
Clusiidae	0±0Aa	0±0Aa	0±0Aa	0±0Aa	0±0Aa	0±0Aa	0±0Aa	0±0Aa	0±0Aa	0±0Aa	0±0Aa	0±0Aa	0±0Aa	0±0Aa	85.86±242.85Aa	0±0Aa	0±0Aa	0±0Aa	0.28±0.8Aa	0±0Aa
Sciaridae	0.16±0.43Aa	0±0Aa	0±0Aa	0±0Ba	0±0Aa	0.05±0.14Aa	0.79±1.77Aa	0±0Aa	0±0Ba	1.97±3.48Aa	0.2±0.48Aa	0.76±1.86Aa	0±0Aa	0±0Ba	0±0Aa	0±0Aa	0±0Aa	0±0Aa	13.11±23.9Aa	0.66±1.97Aa
Chironomidae	0.08±0.22Aa	0.6±1.03Aa	21.12±39.78Aa	22.44±59.33Aa	39.96±83.1Aa	0±0Aa	0.3±0.68Aa	2.54±5.44Aa	2.05±2.95Aa	3±7.28Aa	0.12±0.29Ab	0±0Ab	2.89±3.35Aab	2.75±5.61Aab	5.77±9.07Aa	1.16±2.67Aab	0.16±0.42Ab	2.93±1.93Aab	7.98±13.72Aa	8.17±14.37Aa
Muscidae	0±0Aa	0.09±0.23Aa	1.78±2.87Aa	2.33±5.04Aa	5.29±12.57Aa	0.03±0.08Aa	0.39±0.66Aa	3.25±8.59Aa	0.05±0.13Aa	0.22±0.57Aa	0±0Aa	0±0Aa	0±0Aa	2.11±4.26Aa	0±0Aa	0±0Aa	0±0Aa	0±0Aa	0.09±0.27Aa	0.08±0.25Aa
Cerambycidae	0.24±0.65Aab	0±0Ab	0.66±1.88Aa	0.3±0.95ab	0±0b	0±0Aa	0.38±0.85Aa	0±0Aa	0±0Aa	0±0Aa	6.00±12.38Aa	0±0Aa	0.71±1.1Aa	0.22±0.58Aa	0±0Aa	1.21±2.97Aa	0±0Aa	0±0Ba	0.47±1.08Aa	0±0Aa
Scolytinae	2.57±6.79Aa	0±0Aa	0±0Aa	0±0Aa	0±0Aa	3.48±9.2Aa	0.63±1.42Aa	0±0Aa	0±0Aa	0±0Aa	0±0Aa	0±0Aa	0±0Aa	0±0Aa	0±0Aa	16.97±33.59Aa	0±0Ab	0±0Ab	0±0Ab	0±0Ab
Silphidae	0.48±0.67Aa	0.18±0.48Aa	0±0Aa	0.47±1.01Aa	0.62±1.95Aa	0.1±0.27Aa	0±0Aa	1.35±2.52Aa	0±0Aa	0.39±1.03Aa	0±0Aa	0±0Aa	0±0Aa	0±0Aa	0±0Aa	0.51±1.44Aa	0.36±0.95Aa	0±0Aa	0±0Aa	0±0Aa
Cryptophagidae	0.16±0.43Aa	0±0Aa	0.39±1.11Aa	0.2±0.64Aa	0±0Aa	0±0Aa	0±0Aa	0±0Aa	0.22±0.59Aa	0.39±1.03Aa	0±0Aa	0±0Aa	0±0Aa	0±0Aa	0±0Aa	0±0Aa	0±0Aa	0±0Aa	0±0Aa	0±0Aa
Buprestidae	0±0Aa	0±0Aa	0±0Aa	0±0Aa	0±0Aa	0±0Aa	0±0Aa	0.52±1.38Aa	0±0Aa	0±0Aa	0±0Aa	0±0Aa	0.17±0.37Aa	0±0Aa	0.07±0.21Aa	0±0Aa	0±0Aa	0±0Aa	0±0Aa	0.15±0.44Aa
Histeridae	0±0Aa	0±0Aa	0±0Aa	0.19±0.60Aa	0±0Aa	0±0Aa	0±0Aa	0±0Aa	0±0Aa	0±0Aa	0±0Aa	0±0Aa	0±0Aa	0±0Aa	0.3±0.84Aa	0±0Aa	0±0Aa	0±0Aa	0.38±1.09Aa	0±0Aa
Curculionidae	0±0Aa	0±0Aa	1.16±3.29Aa	0±0Aa	0±0Aa	0±0Aa	0±0Aa	0.21±0.56Aa	0±0Aa	0±0Aa	0.36±0.89Aa	0±0Aa	0±0Aa	0.11±0.28Aa	0±0Aa	0±0Aa	0.17±0.46Aa	1.11±2.31Aa	0±0Aa	0.07±0.22Aa
Mycetophagidae	0±0Aa	0±0Aa	0.12±0.34Aa	0±0Aa	0±0Aa	0±0Aa	0±0Aa	0±0Aa	0±0Aa	0±0Aa	0±0Aa	0.11±0.28Aa	0±0Aa	0±0Aa	0.16±0.46Aa	0±0Aa	0±0Aa	0.13±0.33Aa	0±0Aa	0.82±2.45Aa
Dermestidae	0.09±0.24Aa	0±0Aa	0±0Aa	0±0Aa	1.55±5.13Aa	0±0Aa	0±0Aa	0±0Aa	0±0Aa	0±0Aa	0±0Aa	0±0Aa	0.11±0.24Aa	0±0Aa	2.93±8.28Aa	0±0Aa	0±0Aa	0±0Aa	0±0Aa	0±0Aa
Elateridae	0.25±0.46Aa	2.15±2.56Aa	3.9±5.2Aa	4.5±3.20Aa	9.38±15Aa	0.79±1.98Aa	0.31±0.43Aa	5.69±10.94Aa	3.83±6Aa	6.43±8.42Aa	1.23±1.92Ab	2.47±2.91Aab	3.13±3.83Aab	3.82±3.7Aab	7.09±10.38Aa	0.44±0.98Aa	0±0Aa	0.25±0.67Aa	1.47±2.36Aa	3.06±5.3Aa
Trogossitidae	0±0Aa	0±0Aa	0±0Aa	0.51±1.60Aa	0±0Aa	0±0Aa	0±0Aa	0±0Aa	0±0Aa	0±0Aa	0±0Aa	0±0Aa	0±0Aa	0.11±0.28Aa	0±0Aa	0±0Aa	0±0Aa	0±0Aa	0.1±0.29Aa	0.22±0.66Aa
Carabidae	0.36±0.59Aa	0.83±1.15Aa	0.34±0.63ABa	1.49±2.04Aa	2.67±3.69Aa	0.75±1.07Aa	0±0Aa	2.6±3.45Aa	1.36±2.58Aa	8.39±12.72Aa	0.47±0.9Aa	0±0Aa	1.9±3.5ABa	0.32±0.84Aa	2.79±5.18Aa	0.91±1.03Aa	0.74±0.99Aa	0±0Ba	2.57±6.11Aa	3.09±5.5Aa
Staphylinidae	1.15±1.81Aa	3.33±4.23Aa	4.15±4.35Aa	4.99±3.80Aa	16.87±21.29Aa	0.03±0.08Ab	0.64±0.6ABab	2.6±3.28Aab	3.99±3.12Aab	13.96±11.9Aa	0.65±0.93Aa	0.11±0.28Ba	4.9±5.31Aa	3.98±6.61Aa	13.85±24.83Aa	1.66±3.08Aa	0.23±0.6Ba	0.84±0.66Aa	2.3±3.02Aa	12.71±19.29Aa
Cucujidae	0.09±0.24Aa	0±0Aa	0±0Aa	0±0Aa	0±0Aa	0±0Aa	0±0Aa	0±0Aa	0±0Aa	0±0Aa	0±0Aa	0±0Aa	0±0Aa	0±0Aa	0±0Aa	0±0Aa	0±0Aa	0±0Aa	0±0Aa	0±0Aa
Scarabaeidae	0±0Aa	0±0Aa	0±0Aa	0±0Aa	0±0Aa	0±0Aa	0±0Aa	0±0Aa	0.24±0.63Aa	0±0Aa	0±0Aa	0±0Aa	0.87±1.95Aa	0±0Aa	0±0Aa	0±0Aa	0±0Aa	0±0Aa	0±0Aa	0.13±0.38Aa
Anthicidae	0.05±0.12Aa	0±0Aa	0.39±1.11Aa	0.51±1.60Aa	1.72±5.7Aa	0.05±0.14Aa	0±0Aa	0±0Aa	0±0Aa	0±0Aa	0.25±0.39Aa	0±0Aa	0.61±1.08Aa	0.25±0.66Aa	0±0Aa	0.17±0.48Aa	0±0Aa	0±0Aa	0±0Aa	0±0Aa

Noctuidae	0±0Aa	0±0Aa	0±0Ba	0.35±1.10Aa	1.55±4.59Aa	0.38±1Aa	0±0Aa	0±0Ba	0±0Aa	0±0Aa	0.66±1.61Aa	0±0Aa	0.98±1.9Aa	0±0Aa	0±0Aa	0.05±0.13Aa	0.17±0.46Aa	0±0Ba	0±0Aa	0±0Aa

Note: Values are presented as mean ± SE. Capital letters indicate differences between woody debris types across different forest types under the same treatment, while lowercase letters indicate differences between woody debris types across different decomposition levels under the same treatment ($p < 0.05$). The invertebrate classification in the table is at the family level, with values representing the species density in woody debris (ind/kg).
